# Anesthetic Management of Progressive Deformity of Tracheal Cartilaginous Sleeve in a Pediatric Patient With Beare–Stevenson Syndrome: A Case Report

**DOI:** 10.1155/cria/6267230

**Published:** 2025-10-16

**Authors:** Yumi Doi, Atsuko Harada, Jiro Tsugawa, Nayuta Higa, Tatsuki Oyoshi, Ryosuke Hanaya

**Affiliations:** ^1^ Pediatric Perioperative Center, Takatsuki General Hospital, Takatsuki, Japan; ^2^ Department of Pediatric Neurosurgery, Takatsuki General Hospital, Takatsuki, Japan; ^3^ Department of Pediatric Surgery, Takatsuki General Hospital, Takatsuki, Japan; ^4^ Department of Neurosurgery, Graduate School of Medical and Dental Sciences, Kagoshima University, Kagoshima, Japan, kagoshima-u.ac.jp; ^5^ Department of Neurosurgery, Ichikikushikino Medical Association Neurosurgery Center, Kagoshima, Japan

**Keywords:** airway management, Beare–Stevenson cutis gyrata syndrome, bronchoscopies, craniosynostosis, tracheal cartilaginous sleeve

## Abstract

Beare–Stevenson syndrome is a rare fibroblast growth factor receptor 2–related disorder characterized by craniosynostosis, midface hypoplasia, cutis gyrata, and developmental delay, with upper airway obstruction being a critical concern in early infancy. Tracheal cartilaginous sleeve is a congenital anomaly associated with fibroblast growth factor receptor 2–related syndromes, which often necessitates tracheostomy because of the potential for significant airway complications. However, serial airway imaging in patients with Beare–Stevenson syndrome with tracheal cartilaginous sleeve has not yet been documented. Our pediatric patient with Beare–Stevenson syndrome underwent airway evaluations using a rigid bronchoscope under general anesthesia at 33 days, 62 days, and then at 3 years of age. The initial rigid bronchoscopy demonstrated tracheal cartilaginous sleeve, and tracheostomy was performed. At 3 years of age, rigid bronchoscopy revealed progressive tracheal cartilage deformity with inward protrusion. This pouch‐like structural change greatly affected anesthesia management because it posed a risk for airway obstruction due to potential tube misplacement. The image resolution of a rigid bronchoscope is superior to that of a flexible bronchoscope, allowing for more precise assessment. Despite the absence of abnormalities on routine flexible bronchoscopy, rigid bronchoscopy provided critical insights into airway changes. Precise imaging allowed multidisciplinary planning, and it highlighted tube malposition as a cause of intraoperative respiratory failure. Tracheal cartilaginous sleeve is a life‐threatening condition that may occur in children with Beare–Stevenson syndrome. This case demonstrated progressive tracheal deformity over time, increasing the risk of sudden airway obstruction and presenting significant anesthetic challenges. Our findings highlight the clinical importance of repeated rigid bronchoscopic evaluations, which provide essential information for safe airway management.

## 1. Introduction

Beare–Stevenson syndrome (BSS, MIM #123790) is a very rare fibroblast growth factor receptor 2 (*FGFR2*)–related craniosynostosis that is characterized by cutis gyrata and developmental delay [1, 2]. It is often associated with midface hypoplasia, leading to upper airway obstruction, which makes the respiratory status and airway management critical to the patient surviving infancy [2].

We report the case of a pediatric patient with BSS for whom we performed airway evaluation with rigid bronchoscopies at 33 days, 62 days, and then at 3 years of age. The initial rigid bronchoscopy demonstrated tracheal cartilaginous sleeve (TCS). Rigid bronchoscopy requires general anesthesia, and its use is typically limited to relatively large facilities. Therefore, it had not been performed for this patient for almost 3 years. Although it is not necessary to perform rigid bronchoscopy routinely, we considered it reasonable to perform rigid bronchoscopy to evaluate the status of the airway while our patient was under general anesthesia for surgical intervention. At the age of 3 years, rigid bronchoscopy had revealed pronounced tracheal deformity before the procedure for foramen magnum decompression. We thought this could have a major impact on intraoperative airway management.

By documenting the progression of TCS over time, we highlight the importance of continuous airway evaluation in patients with BSS, providing insight into airway management in patients with BSS that develop TCS.

## 2. Case Presentation

**Figure 1:**
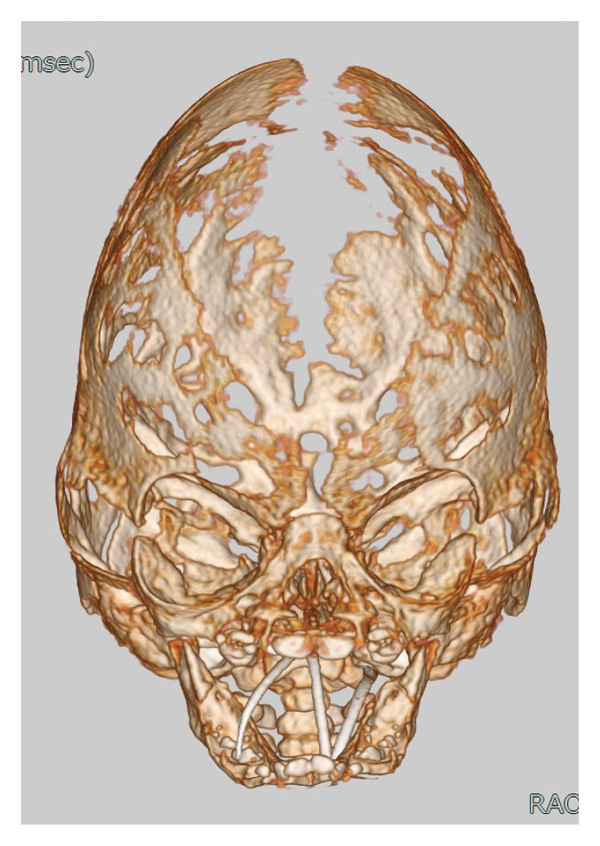
Head three‐dimensional reconstructed CT image (day 26). Bilateral coronal and lambdoid sutures are fused, with a markedly enlarged anterior fontanelle. The skull exhibits a cloverleaf‐like deformity with thinning of the bone, suggesting increased intracranial pressure.

A 3‐year‐old girl (height: 81 cm, weight: 7.3 kg) was admitted for foramen magnum decompression due to progression of tonsillar herniation. She had been prenatally diagnosed with craniosynostosis and was delivered at 35 weeks and 2 days of gestation at another institution, weighing 2829 g with Apgar scores of 8 and 9 at 1 and 5 min, respectively. In addition to bilateral coronal and lambdoid craniosynostosis, she presented with a cloverleaf‐like skull, bulging anterior fontanelle, proptosis, maxillary hypoplasia, cleft soft palate, perineal skin defects, and broad toes (Figure 1). These findings were initially thought to suggest Pfeiffer syndrome.

Due to respiratory distress secondary to upper airway obstruction and tracheomalacia, the patient was admitted to the neonatal intensive care unit and she received high‐flow nasal support. However, as hydrocephalus progressed, neurosurgical intervention was required. Her trachea was intubated at 23 days old due to unstable airway patency and in preparation for long‐distance hospital transfer. She subsequently underwent bilateral coronal suturectomy and rigid bronchoscopy at 33 days old in which TCS was diagnosed (OP1) (Figure 2(a)), bilateral lambdoid suturectomy with foramen magnum decompression at 50 days old (OP2), and tracheostomy and another rigid bronchoscopy at 62 days old (OP3) (Figure 2(b)) before being transferred back to a local hospital near her home.

Figure 2Rigid bronchoscopy images. White arrow: the left edge of the tracheal cartilage. (a) Day 33. No signs of stenosis or obstruction were observed in the supraglottic, glottic, or subglottic regions. The upper to mid‐trachea exhibits a tracheoesophageal membrane, yet it maintains a cylindrical shape, with the fused tracheal cartilaginous rings. In the mid‐trachea, overlapping of the tracheal cartilage rings is observed. (b) Day 62. The findings were consistent with the tracheal cartilaginous sleeve observed on day 33. (c) 3 years old. Upon removal of the tracheostomy tube and subsequent rigid bronchoscopy, the protrusion of the left tracheal cartilage end into the lumen observed in (a) and (b) had become more pronounced. This region could only be visualized after removing the tracheostomy tube.

**Figure:**
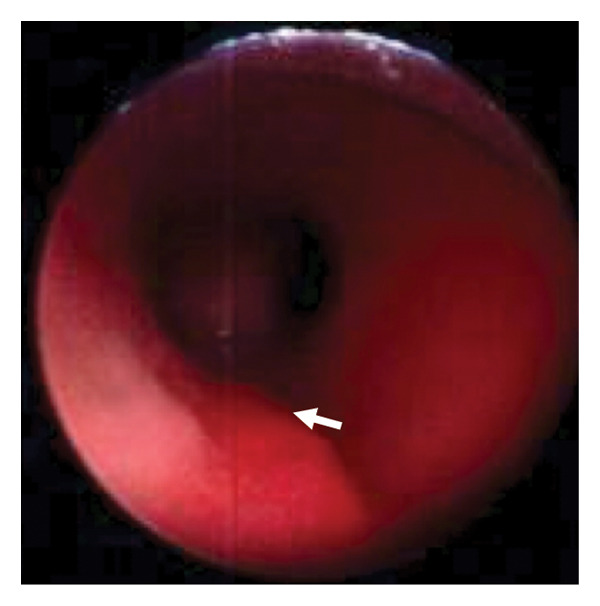
(a)

**Figure:**
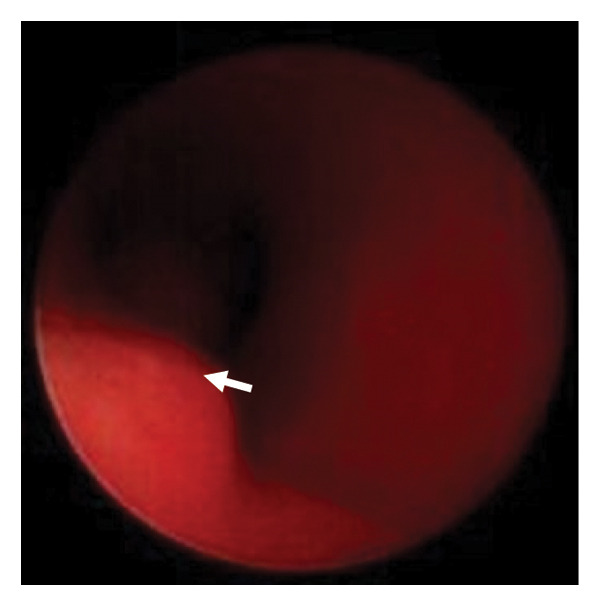
(b)

**Figure:**
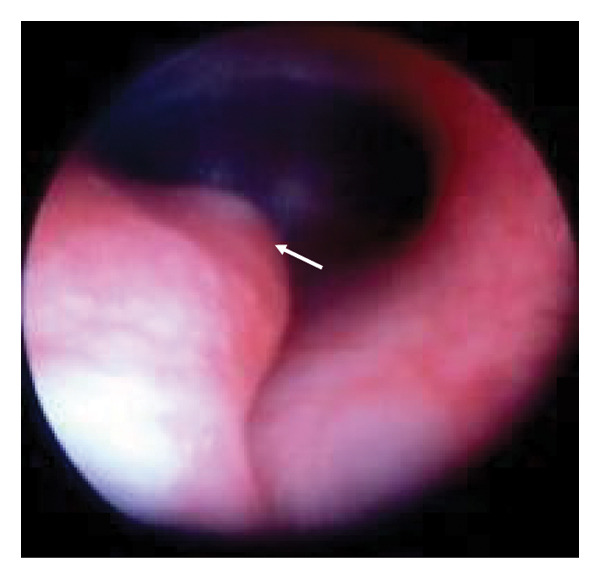
(c)

The otolaryngology department at that hospital managed her tracheostomy tube, and a ventriculoperitoneal shunt was placed at 6 months of age to treat hydrocephalus. Genetic testing revealed a heterozygous *FGFR2* Y375C mutation, which confirmed the diagnosis of BSS, but there had been no significant family history.

The patient continued follow‐up at the departments of neurosurgery and otolaryngology. She was dependent on a tracheostomy tube (Bivona tracheostomy tube, inner diameter: 3.5 mm, NEO Straight Flange, Smith‐Medical Japan, Tokyo, Japan), without a ventilator or oxygen supplementation, and she received enteral nutrition via a nasogastric tube. Tracheostomy tube changes were recorded to have been performed regularly without any difficulties, and flexible bronchoscopy assessments indicated no formation of granulation tissue in the airway. However, tonsillar herniation progressed, and she was readmitted to our institution for foramen magnum decompression.

**Figure 3:**
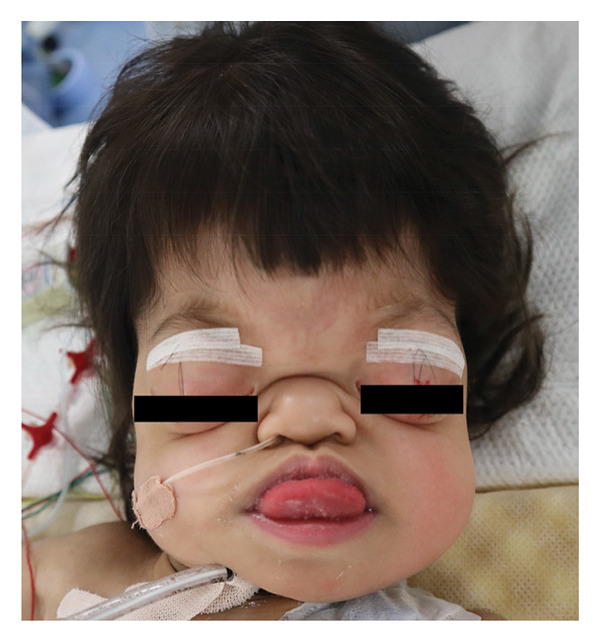
Characteristic facial features at 3 years of age. After anesthesia induction, a plastic surgeon performed temporary eyelid closure with sutures.

On readmission to our hospital, the patient’s SpO_2_ was 94% in room air with spontaneous breathing via tracheostomy tube. Her characteristic craniofacial features included ocular proptosis, a narrow and protruding forehead, midface hypoplasia, macroglossia, and cutis gyrata, which had been unclear at birth but had gradually become apparent (Figure 3). Neurodevelopmental delay was noted. She could roll over and sit with assistance but was unable to stand or walk on her own. She could respond to simple verbal commands, such as giving objects when requested.

For the tonsillar herniation surgery, the patient entered the operating room with her mother without premedication for neurophysiological monitoring. After standard monitoring was applied, anesthesia was induced by propofol and fentanyl via a preestablished intravenous catheter. After confirming ventilation via tracheostomy tube, muscle relaxant was administered. Due to the presence of a tonsillar hernia, the patient was carefully positioned to avoid excessive flexion of the neck, and a rigid bronchoscope was inserted to observe the subglottic area, which revealed a small cyst. After sufficient oxygenation in preparation for apnea for a short duration, we removed the tracheostomy tube and advanced a rigid bronchoscope from the tracheostomy to the carina. Rigid bronchoscopy revealed substantial inward protrusion of the left tracheal cartilage edge (Figure 2(c)). The tracheostomy tube was replaced with a Microcuff pediatric endotracheal tube (inner diameter: 4.0 mm, Avanos Medical Inc., USA), and flexible bronchoscopy confirmed appropriate placement without tube migration into the pouch‐like region created by this cartilage deformity. The tube was secured using tape and dressings.

Additional intraoperative challenges included difficult intravenous access requiring central venous catheterization, eyelid suturing to prevent exposure keratopathy, facial pressure ulcer prevention, and setup for motor‐ and somatosensory‐evoked potential monitoring. Intraoperative arterial blood gas analysis indicated stable ventilation, although glucose supplementation was required for mild hypoglycemia. After surgery, the tracheal tube was switched back to the Bivona tracheostomy tube and the patient was transferred to the pediatric intensive care unit.

## 3. Discussion

To the best of our knowledge, this is the first report to document the natural course of tracheal cartilage deformity through serial imaging and to describe anesthesia management in a patient with BSS who developed TCS.

In this case, rigid bronchoscopy images were taken in early infancy and then at 3 years of age, which demonstrated progressive tracheal cartilage deformity (Figure 2). However, this unique progress is limited to our single case and it is unclear how other cases of BSS will progress. This syndrome itself is rare, with a limited number of clinical case reports, and it is unclear how often TCS is accompanied by BSS, or how TCS of BSS progresses differently from TCS with other *FGFR2*‐related syndromes. Further examination of the airways of patients with BSS is therefore required, with accumulation of more cases and the exploration of various imaging modalities.

BSS is distinct from other *FGFR2*‐related craniosynostoses, such as Apert, Pfeiffer, and Crouzon syndromes, based on clinical manifestations and genetic mutations [3]. To date, the identified mutations associated with BSS are *FGFR2*, S372Y, and Y375C [4–6]. This patient was initially suspected to have Pfeiffer syndrome from her clinical features because cutis gyrata (which is indicative of BSS) was not apparent in infancy. However, genetic testing conducted at the previous hospital revealed the Y375C variant of *FGFR2*, and the patient was diagnosed with BSS. In these cases of syndromic craniosynostosis accompanied by midface hypoplasia and malformation, the upper airway (including the oral cavity, nostrils, nasal cavity, and pharynx) is anatomically narrow. If the patency of the upper airway cannot be maintained, airway intervention such as tracheostomy is often necessary from the neonatal period to early infancy. Despite tracheostomy, patients with BSS have a poorer prognosis than those with other *FGFR2*‐related syndromes [7], with TCS being a key factor.

TCS is a congenital anomaly characterized by an abnormal formation of the tracheal cartilage. Unlike the normal trachea, which consists of a series of distinct, C‐shaped cartilaginous rings separated by membranous gaps, TCS presents as a continuous, rigid cartilaginous structure. Furthermore, TCS is sometimes an undetected source of morbidity and mortality in patients with craniosynostosis syndromes with *FGFR2* mutation such as Pfeiffer syndrome, Apert syndrome, Crouzon syndrome, and BSS. This airway anomaly has not been widely reported among patients with syndromic craniosynostosis. Among these craniofacial syndromes, Pfeiffer syndrome and BSS are thought to be particular risks of TCS, but the true incidence of TCS is largely unknown [8, 9]. Notably, however, tracheostomy is necessary in patients with BSS for them to survive infancy. Even if a tracheostomy is performed, the secretion function of the mucosa and passive immunity of the airway are impaired if the patient has TCS. As many as 90% of patients with craniosynostosis accompanied by TCS therefore die before the age of 2 years, with 58% of these deaths being due to respiratory distress [10]. Formation of granulation in the trachea and posterior tracheal injury also require attention [8]. TCS is plate‐like, so it is rigid and is prone to granulation tissue formation due to contact with the tip of the tracheostomy tube. Tube malposition is another concern; in one reported case, tracheal deformity due to TCS led to a sudden ventilatory impossibility in which a tracheostomy tube became trapped in a false cavity in the trachea, resulting in airway obstruction and sudden death [11].

TCSs are often only found incidentally at autopsy because they are difficult to diagnose without direct visualization of the trachea [10]. Diagnosing TCS might be possible by flexible fiberoptic bronchoscopy despite the poor quality of images, but it is considered to be unsuitable for detailed observation. Direct visualization using a charge‐coupled device camera such as a rigid bronchoscope is therefore recommended for accurate diagnosis and precise evaluation of the airway.

We performed airway evaluations using a rigid bronchoscope on this patient twice in early infancy and once more at 3 years. Compared with the initial evaluation, the left tracheal cartilage edge had significantly protruded into the tracheal lumen by the third time (Figure 2(c)). Otolaryngologists who managed this patient’s tracheostomy reported the absence of granulation tissue in the airway according to their assessment using a flexible bronchoscope during each tracheostomy tube replacement. However, the pouch‐like or wave‐like region created by this cartilage deformity was difficult to observe while the tracheostomy tube remained in place. Previous reports of sudden death due to airway obstruction suggest that misplacement of the tube into such a pouch‐like structure could result in ventilatory failure [11].

The surgical site was located in the occipital region, so the patient had to be placed in a prone position, which would create difficulties in intraoperative management of ventilation failure or tube disposition. Although the reports from otolaryngologists had indicated no abnormalities, we initially hesitated to perform a rigid bronchoscopy assessment in a hyperextended position for this patient because of tonsillar herniation. However, had the endotracheal tube been blindly exchanged, it is possible that ventilation failure might have occurred, depending on the alignment between the tracheal axis and the bevel position of the tube. Consequently, accurate assessment of the airway condition before the procedure and confirmation of the tube position after exchange were thought to contribute to safe intraoperative respiratory management.

We propose that even outside of surgical contexts, clinicians should consider the possibility of tube migration into pouch‐like regions caused by cartilage deformity in patients with BSS with tracheostomy who have sudden respiratory failure. Notably, we were able to obtain this knowledge through these serial image evaluations, and we suggest the importance of recognizing the risk of tube migration in case of sudden deterioration.

## 4. Conclusion

Serial rigid bronchoscopy images, performed in a patient with BSS that had developed TCS, revealed progressive cartilage deformity. Because BSS is extremely rare and rigid bronchoscopy is infrequently performed, no previous reports have documented consecutive airway images, so the natural progression of airway changes remains poorly understood. Importantly, sudden and life‐threatening airway obstruction can occur despite the presence of a tracheostomy tube due to severe deformity of the tracheal cartilage. This case underscores the importance of thorough preoperative airway assessment and meticulous attention to tube placement in patients with BSS and TCS.

**Nomenclature**
BSS: Beare–Stevenson cutis gyrata syndrome
*FGFR 2*
: Fibroblast growth factor receptor 2
TCS: Tracheal cartilaginous sleeve

## Ethics Statement

The authors have nothing to report.

## Consent

We obtained written consent for publication from the patient’s parents.

## Conflicts of Interest

The authors declare no conflicts of interest.

## Author Contributions

Yumi Doi is the attending anesthesiologist and wrote the manuscript. Atsuko Harada, Nayuta Higa, and Tatsuki Oyoshi performed the neurosurgery. Jiro Tsugawa performed rigid bronchoscopy and made a decision regarding airway management. Ryosuke Hanaya oversaw the manuscript.
